# The role of intraspecific trait variation in driving post‐metamorphic survival: Implications for recruitment in open populations

**DOI:** 10.1002/ece3.70065

**Published:** 2024-08-06

**Authors:** Luis Giménez, Stuart R. Jenkins

**Affiliations:** ^1^ School of Ocean Sciences Bangor University Menai Bridge UK; ^2^ Biologische Anstalt Helgoland, Alfred Wegener Institut Helmholtz‐Zentrum für Polar Und Meeresforschung Helgoland Germany

**Keywords:** body size, competition, intraspecific phentypic variation, Jensen's inequality, open populations, recruitment, settlement

## Abstract

Most ecological studies attempting to understand causes of population dynamics and community structure disregard intraspecific trait variation. We quantified the importance of natural intra‐cohort variation in body size and density of juveniles for recruitment of a sessile marine organism, the barnacle *Semibalanus balanoides*. Barnacles are representative of species organised in metapopulations, that is, as open local populations connected by larval dispersal. We tracked the individual growth and survival of a cohort of juvenile barnacles from two shores of North Wales. Barnacles settled as larvae in spring of 2002 on previously cleared rock. The density of these new recruits was experimentally manipulated in June and randomly selected individuals were monitored from June to October to evaluate the role of barnacle size and density in predicting survival. In doing so we characterised density at three spatial scales (quadrat: 25 cm^2^, cells within quadrats: 25 mm^2^ and neighbourhood: number of neighbours in physical contact with the target barnacle). At all scales, variations in juvenile body size exacerbated the effect of density‐dependent mortality on population size. While density‐dependent mortality was very intense in the small‐sized individuals, large‐sized individuals experienced very weak density‐dependent mortality and showed high survival rates. Using the concept of ‘Jensen inequality’, we show that important biases in estimations of survival, based on population size only, occur at high barnacle densities, where survival is low. Our study highlights the role of body size variation in understanding dynamics of open populations.

## Graphical abstract

Both theory and studies of open populations disregard the role of intraspecific trait variation as driver of recruitment and population dynamics. We show the prevailing influence of body size in driving density‐dependent survival in two populations of intertidal barnacles. These results have important implications for the further development of theory of open populations.

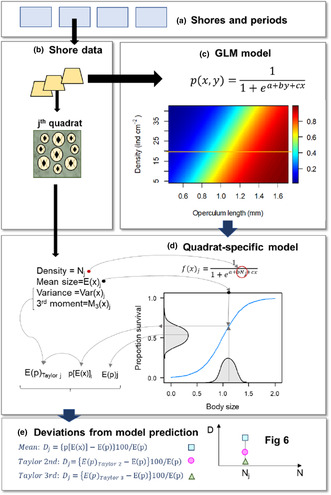

## 1 INTRODUCTION

A central question in ecology concerns the understanding of mechanisms driving population dynamics and community structure. Most approaches to address these questions ignore intraspecific trait variation (ITV), except for the role of age, stage and size among cohorts (through, e.g., age and stage distribution models: Caswell, 2001). The lack of information on the importance of ITV, within a given cohort, is striking given that phenotypic variation is considered the stuff of evolution (West‐Eberhard, 2003). However, in the past years, a growing body of literature, mostly on plants, has uncovered several processes by which variation in morphological and physiological traits can drive population dynamics, community structure and ecosystem function (Bolnick et al., 2011; Des Roches et al., 2018; Stump et al., 2022). For instance, the intensity of interspecific competition can be modulated by the body size of the neighbouring species (Cameron et al., 2019) and population dynamics can be affected by intraspecific differences in the efficiency of competition for resources (Stump et al., 2022; Zaiats et al., 2021).

Understanding the role of ITV in population dynamics is central to the development of conservation efforts in the light of climate change (Moran et al., 2016; Violle et al., 2012). Increasing temperatures are expected to lead to a reduction in the fundamentally important trait of body size (Gardner et al., 2011; Lindmark et al., 2018; Ohlberger, 2013) with implications for organismal performance (Altwegg & Reyer, 2003; Marshall et al., 2018; Rowe & Ludwig, 1991). In addition, exploitation of populations tends to differentially target individuals from large size classes leading to changes in size distribution (Audzijonyte et al., 2013; Xu, 2016). However, at the intracohort level, most ecological studies and ecological theories of population dynamics still focus on numerical effects. This is the case for the theory of open populations (Caley et al., 1996; Hixon et al., 2002) where dynamics are driven by the balance between arrival of propagules to the local habitat and subsequent density‐dependent processes (i.e. defined as pre‐ vs. post‐settlement processes, respectively).

**FIGURE 1:**
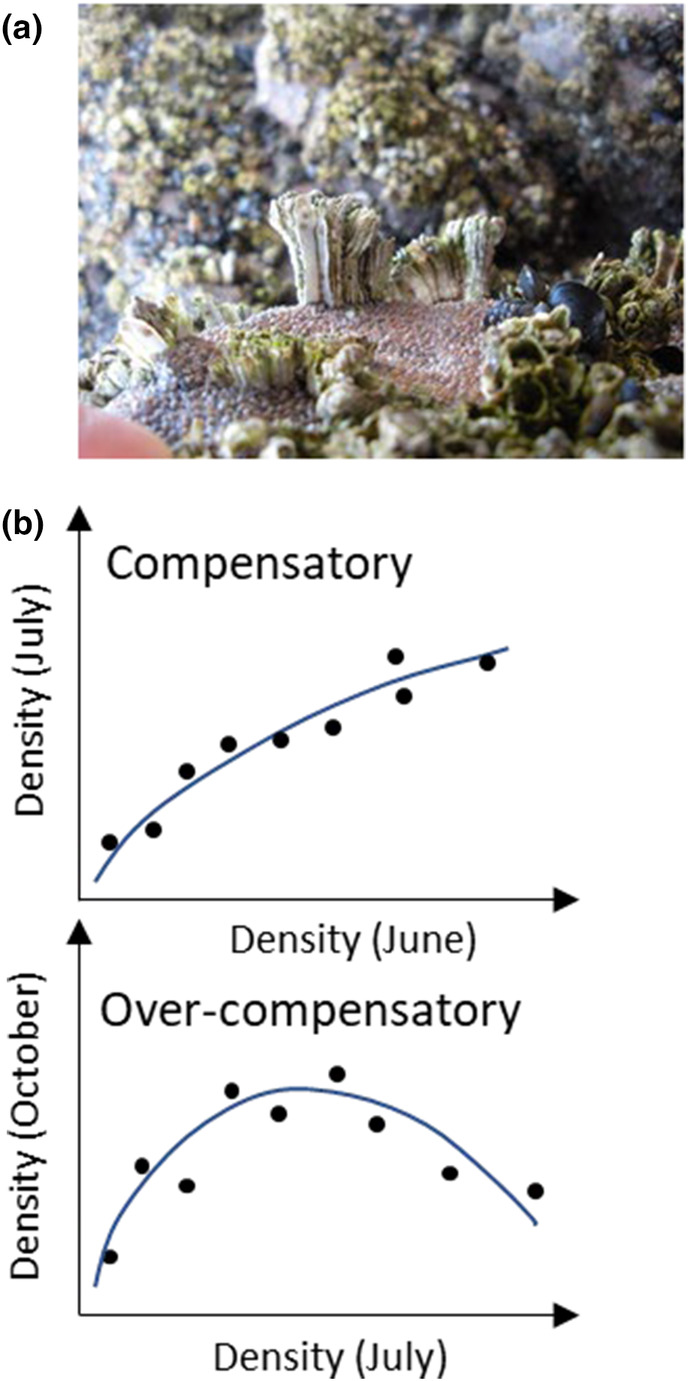
(a) Image of barnacles that have grown in high densities, forming hummocks and taking a columnar shape. Most of the hummock has been lost, potentially through wave action, leaving a few survivors. (b) Shift in the recruitment function from compensatory (June vs. July densities) to overcompensatory (July vs. October densities) where high barnacle density results in extremely high mortality (summarised from Jenkins et al., 2008).

Open populations are found in organisms with complex life cycles, including many marine bottom invertebrates, anurans and aquatic insects. For open marine populations, there are only a handful of studies evaluating the role of ITV in ecological processes including predation and competition (e.g. Cameron et al., 2019; Gribben et al., 2020; Griffin & Silliman, 2018; Hedge et al., 2014; Smee et al., 2013; Toscano & Griffen, 2012). Whether a paradigm shift is needed depends on the extent to which phenotypic variation is important in driving population dynamics. Here, we use populations of a marine barnacle (*Semibalanus balanoides*) as a model system to quantify the importance of intraspecific variation in body size as a driver of population size. *S. balanoides* has a wide distribution, occupying the intertidal rocky shore spanning the Atlantic coast of the USA and Canada, and northern Europe. Marine barnacles have been used for decades as textbook examples of inter‐ and intraspecific competition, leading to important contributions towards understanding drivers of population dynamics and community organisation (Begon et al., 2006; Ricklefs & Miller, 1999). Competition is an important source of mortality (Barnes & Powell, 1950; Bertness et al., 1998; Connell, 1961; Jenkins et al., 2008). Crowding leads to columnar growth and the formation of hummocks, dome‐like structures that increase mortality risk through wave action (Figure 1a). Barnacle body size varies across several spatial scales (Burrows et al., 2010) and barnacle density is a major driver of growth (Crisp, 1960; Wethey, 1983). However, population size in barnacles is frequently characterised by percentage cover (i.e. the product of density and body size) and individual body size is often ignored. In general, there has been little interest in quantifying body size effects on population dynamics (but see Wethey, 1983).

We quantified the effect of barnacle density and body size in explaining survival in two local populations from North Wales at two different times in the summer season. The quantification was carried out at three spatial scales: (1) quadrat scale: 25 cm^2^, (2) cell scale: 25 mm^2^ and (3) neighbourhood scale (i.e. individuals in contact with the target barnacle). The quadrat scale is the one usually employed by ecologists to quantify barnacle density, using quadrats of 5 × 5 cm. However, the density of barnacles experienced by a single individual may be decoupled from the density quantified at the quadrat scale because of variations in spatial aggregation. Individual barnacles are likely to interact most strongly with individuals located within a few millimetres of distance, either through physical contact with their exoskeletons or through interactions with the feeding apparatus. This was captured by setting a small quadrat of 5 × 5 mm centred on the target barnacle. At the quadrat scale, Jenkins et al. (2008) used the study populations to demonstrate that the strength of density‐dependent mortality can change over time, from compensatory (Figure 1b; see also Jenkins et al., 2008, Figure 1) in early summer to overcompensatory in late summer to autumn (Figure 1c). In addition, Gimenez and Jenkins (2013) showed that barnacle growth explained well the switch to overcompensation by limiting the available space, despite initial juvenile mortality. Here, we explicitly addressed biases in estimation of recruitment to the adult population committed by ignoring the importance of body size variation and evaluated the hypothesis that barnacle body size drives survival in addition to the known effect of barnacle density.

## 2 METHODS

### 2.1 Sampling barnacles

We evaluated the role of density and body size from data collected by Jenkins et al. (2008) in their study of post‐settlement density dependence. These authors studied barnacle recruitment at two rocky intertidal shores located in Anglesey 2 km apart (south of Porth Cwyfan: 53°11·2′ N, 4°30·0′ W; 53°10·7′ N, 4°29·2′ W, respectively). At each shore, they defined 70 small quadrats of 5 × 5 cm size within the middle of the zone of barnacle distribution along 100 m shoreline, in areas devoid of macroalgal canopy. The rock surface for each quadrat was cleared in April 2002 and settlement was allowed to occur between April and the end of May. In June, quadrats with high barnacle density (>30 cm^−2^) were selected (total = 54) and the density of recent settlers (~ 1 month old) was manipulated by removing individuals at random. Survival was then monitored, initially after approximately 1 month (July 2002) and thereafter approximately every 4 months (October 2002, February 2003 and June 2003) through the use of digital images. The original study by Jenkins et al. (2008) used average densities and the proportion of survivors per quadrat; in a subsequent analysis, Gimenez and Jenkins (2013) estimated the average barnacle size per time and modelled the role of body size and barnacle growth in explaining temporal changes in post‐settlement survival.

The present analysis is based on two data sets; the first set corresponds to average barnacle densities per quadrat as used by Jenkins et al. (2008) while the second set consists of estimations of body size and the fate of individual barnacles (survivor or dead) obtained from a new survey of the same images. This new data set records the body size and fate of individuals from 2460 individual barnacles selected at random (shore 1: 30 individuals from 44 quadrats = 1320 individuals; and shore 2: 30 individuals from each of 37 quadrats = 1110 individuals) over two periods, June–July and July–October. The strongest density‐dependent interactions occurred between June and October; after October, variations in barnacle density dropped because few individuals remained in several of the quadrats with high initial densities (Jenkins et al., 2008). Not all quadrats were used (initial total = 56 per shore): quadrats with very low density (density < 3.9 ind cm^2^) were discarded; an additional quadrat was lost from shore 1.

The 30 target barnacles within each quadrat were selected by defining a grid of eighty 5 × 5 mm cells and sampling 30 of these at random. Within each cell, individuals were given random numbers and a single individual per cell was randomly selected and measured. This ensured that individuals were not chosen according to size. For instance, size bias could occur if barnacles at the centre of the cell were to be chosen because larger barnacles occupy more space within the cell. For each of the 30 barnacles in each quadrat, the density of neighbours that they experience was calculated at three different scales. At the **quadrat** scale, the overall quadrat density was used. At the **cell scale,** we centred a quadrat of 5 × 5 mm upon each target barnacle and counted the number of individuals within that cell. In a few cases, the process of random selection led to choosing barnacles that were in adjoining cells and were in close proximity to each other. In such cases, we randomly re‐selected another barnacle of one of those cells to ensure that target individuals were not in the same cell. Here, for the period July–October and at high densities, the exoskeleton of a fraction of the dead barnacles remained intact: in those cases, we quantified the number of living and dead barnacles and considered those in the statistical models (see below). At the **neighbourhood scale,** the number of living and dead barnacles with exoskeletons in physical contact with the target barnacle was used as the estimate of neighbour density.

The size and fate (survived or died) of all target barnacles within all quadrats were monitored at each time interval using the freeware ImageJ. Following the approach of Jenkins et al. (2008), the length of the operculum was used as the measure of size. Two measurements were taken (anterior–posterior and perpendicular to it) and averaged. The size of the operculum defines the area available for feeding, respiration and reproduction, and unlike basal diameter, shows little covariation with barnacle density (Gimenez & Jenkins, 2013), hence meeting the requirements of GLM (Zuur et al., 2009).

### 2.2 Statistical analysis

The importance of barnacle body size and average density per quadrat was evaluated using GLM or GLMM (see below) where the response variable, the fate of a single individual, is modelled from a binomial distribution. GLM model selection and comparison were performed in R through the MASS package (Venables & Ripley, 2002), while for GLMM, the package lme4 was used. Models were fit using a logit link function leading to the following equation:

(1) $$p\left(S,D\right)=\frac{1}{1+e^{-f\left(S,D\right)}}$$

In Equation 1, *p(S,D)* is the survival probability, depending on body size (S), density or number of neighbours (D); and *f(S,D)* represents a linear model that relates the predictor variables to the link function, *logit (p)* = *f(S,D)*. The initial linear model, *f(S,D)*_*initial*_, included an interaction between barnacle density and body size; subsequent models included only the additive term and terms for either density or body size (Equation 1). Model selection was carried out using the Akaike information criteria (AIC). Comparisons of different models were carried out with AIC as follows: (1) if ΔAIC >3, then the most parsimonious model was selected; (2) if ΔAIC ≤3 and the most parsimonious model had the lower AIC, then we selected the most parsimonious model. However, (3) if ΔAIC ≤3 but the most complex model showed the lowest AIC, then we used likelihood ratio test with the ‘anova’ function (based on the Chi‐square distribution) to determine if the most complex model contained a significant term (at *α* = .05). Most of the best models contained both body size and density as predictors. Model validation and fit were evaluated using the package DHARMa (Hartig, 2022): residuals did not show any evidence of bias, issues with dispersion or deviations from the assumption of binomial distribution.

For the quadrat scale, there was only a single value of barnacle density per quadrat and hence the GLM did not contain any random term. We first checked the correlation between density and body size which was very low for both shores (Figures S1, and S2). Here, all available data in subsequent analyses and models were fully validated (Figure S3). For the cell and neighbourhood scales, there were several values of barnacle density (or number of neighbours per quadrat). Therefore, we used GLMM with ‘quadrat’ incorporated in the model as a random factor, and the full model was coded as P ~ D + S + D:S+(1|Quadrat), where P is the proportion of survivors, D is the barnacle density (or number of neighbours) and S is the barnacle operculum length. For models fitting survival between July and October, we ran separate models with density quantified as number of living individuals only and the sum of living and dead individuals (exoskeletons). In both cases, the space of predictors was not fully covered and attempts at model fitting resulted in singular fit or failure to convergence. We therefore ran the models over a restricted range covering the region where the space of predictors was fully covered (Figures S1 and S2). Within those ranges, models were fully validated (Figures S4 and S5).

We compared the contribution of body size and density (or number of neighbours) to explain survival through two different approaches, that is, the parameter estimates and the percentage variance explained by each variable. This comparison was carried out after fitting additive models to the normalised predictor variables. Normalisation (=mean subtraction and division by the standard deviation) results in equally scaled predictors and unit‐less parameter estimates. Additive models were used here to provide information for comparison only: in one exception, the best model contained the interactive effect, but it explained only a very small percentage of variation (see results); in addition, shore 2 survival between June and July was explained only by barnacle size, but the additive model provides values of the contribution of density that is near zero, which were used for plotting. For the quadrat scale, the per cent contribution to explaining the total variation was calculated from the deviances of each separate predictor. For the cell and neighbourhood scales, we calculated the contribution, as marginal pseudo‐*R* ^2^, using the methods defined in Nakagawa and Schielzeth (2013) and the package *partR2* (Stoffel et al., 2021). The marginal contribution does not account for the variance associated with the random variation, which is then considered as ‘unexplained’. Density and body size explained a small percentage of the total variance. However, this is a characteristic property of binomial models because observations can only acquire values of 0 or 1 while fitted values will lie within the range of 0, and 1 without reaching those limits (such values do not belong to the set of numbers given by the logistic function).

### 2.3 Jensen gap

**FIGURE 2:**
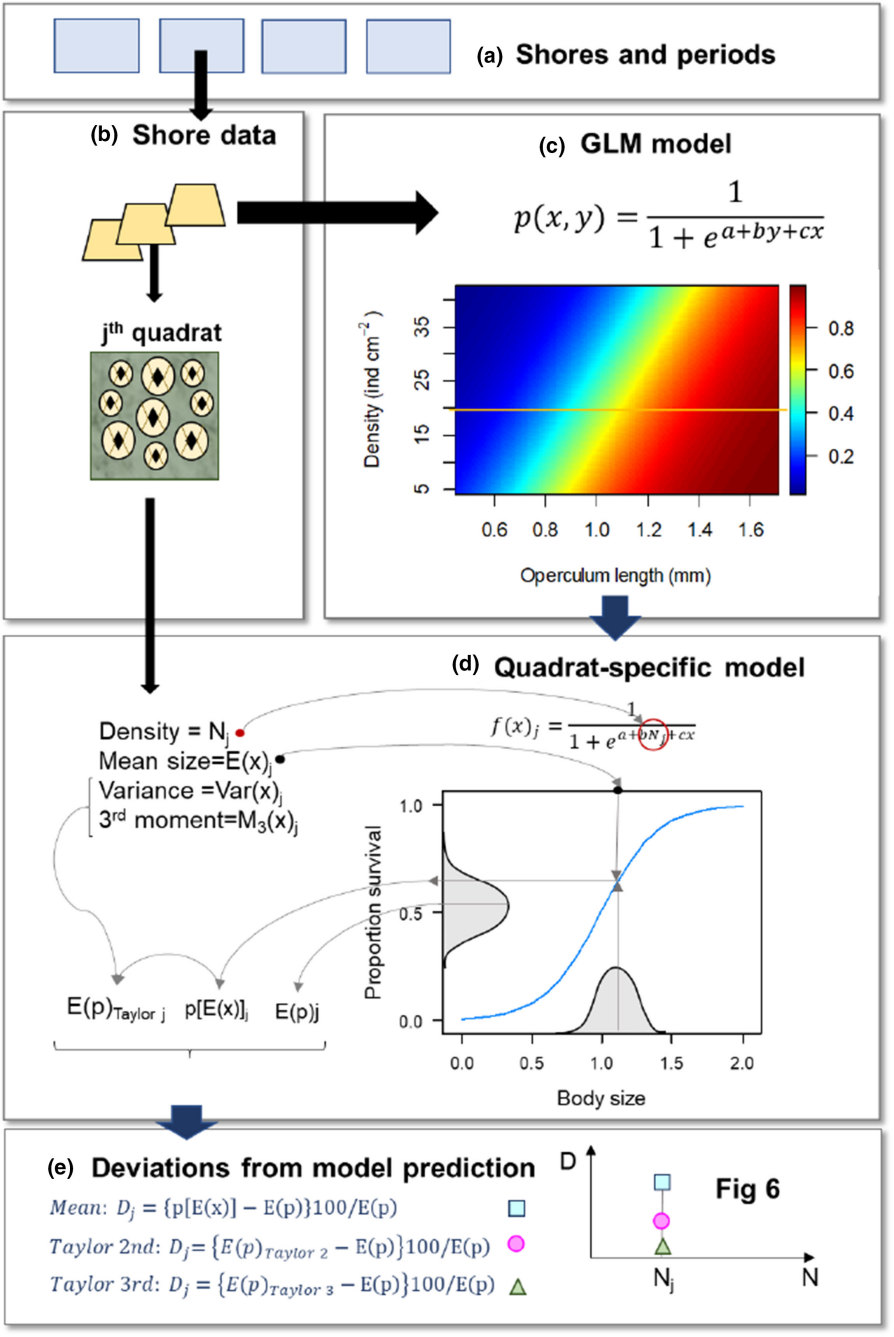
Summary of calculation of the Jensen gap. (a) Data from each shore and time period are used. (b,c) For each combination of shore and time period, a statistical model is fitted with barnacle body size and density as predictors (2D image). (d) By plugging the barnacle density recorded at a given quadrat, we obtain a quadrat‐specific model. In the 2D image in (c), any such model covers a horizontal line, where barnacle density is constant (one such line is drawn as an example). The mean barnacle size is plugged into the model: Variance and third‐order moments are calculated for each quadrat and used to calculate the expected survival probability as Taylor approximations. (e) Taylor approximations and fitted survival probabilities are used to calculate the Jensen gap. Each quadrat produces a single point per approximation (i.e. 3 points).

For the quadrat scale, we used Jensen's inequality to evaluate the importance of variation in body size in obtaining estimations of barnacle survival (Figure 2). A critical issue associated with the observed variation in body size is that estimations of survival (for each value of barnacle density) based on the average barnacle size as a predictor are biased, unless the function, *f(x)*, relating survival and body size adopts very specific forms (e.g. is linear) or the variance in body size is zero. Because such functions were non‐linear and body size varied at each value of barnacle density (see results), the estimations of average survival are biased as described by the so‐called Jensen inequality (Bolnick et al., 2011). Here, we used several approximation methods to estimate the combined role of the non‐linear response to density and the variation in body size, using information on the variance and the skewness of the size distribution (see below). We applied our approach separately to the three cases where the best model retained both body size and barnacle density as predictors (shore 1, both periods; and shore 2: July–October: see results).

The Jensen inequality (Bolnick et al., 2011) is expressed as follows:

(2) $$f\left[E\left(x\right)\right]\neq E\left[f\left(x\right)\right]$$

In Equation 2, *x* is body size, *E(x)* is the average barnacle size and *E[f*(*x)]* is the expected survival probability. Applied to our case, Equation 2 indicates that the survival of an average‐sized barnacle does not necessarily give a correct estimation of the average survival of the local population. In such a case, one can obtain an approximation to the average survival by applying expectations to the Taylor approximation of the function *f*(*x*) around the average body size. When such function is approximated to the third order, we obtain:

(3) $$E\left[f\left(x\right)\right]\approx f\left[E\left(x\right)\right]+\mathrm{Var}\left(x\right)∙f^{\prime \prime }\left[E\left(x\right)\right]/2+M_{3}\left(x\right)∙f^{\prime \prime \prime }\left[E\left(x\right)\right]/6$$

In Equation 2, *Var(x)* is the variance in barnacle body size and *M*_*3*_ *(x)* is the third‐order moment, which captures the degree of skewness in the distribution; *f“[E(x)]* and *f”'[E(x)]* are, respectively, the second‐ and third‐order derivatives of *f(x)*, evaluated at the average body size. Hence, one can compare the expected survival probability, predicted by our fitted model, with different approximations, based (1) only on the average barnacle size (hereafter called ‘Mean based approximation’), (2) on the average and variance (Taylor 2nd) and (3) on the average and variance and skewness (Taylor 3rd). Such a comparison helps to understand the contribution of different aspects of intraspecific variation (variance and skewness).

We carried out calculations separately for each quadrat *(*j = 1, …*n)*; therefore, for each quadrat (Figure 2b), we calculated the three different predictions of survival. First, we used the fitted statistical models (Figure 2c) to obtain tile‐specific functions, *f(x)*_*j*_ (Figure 2d), by plugging the tile‐specific barnacle density value *(N*_*j*_ *)* as a known constant:

(4) $$f{\left(x\right)}_{j}=\frac{1}{1+e^{-g\left(N_{j}x_{j}\right)}}$$

In Equation 4, *g(N*_*j*_, *x*_*j*_ *)* = *a + b∙N*_*j*_ *+ c∙x*_*j*_ *+ d∙x*_*j*_ *∙N*_*j*_, where *a*, *b* and *c* are parameter estimates of the model. There is one such model per combination of shore and time period. In most cases, d *= 0*; in consequence, for a given tile‐j, the derivative *dg/dx = a + b∙N*_*j*_ *+ c = k*_*1*_. In one of the models (shore 1, June), the interactive term was retained and *dg/dx = a + b∙N*_*j*_ *+ c + d∙N*_*j*_ *= k*_*2*_. In the next paragraph, we refer to those constants as *k*_*l*_ with *l = 1*,*2* depending on the model.

The plot of Figure 2d shows graphically the bias introduced by using the mean‐based approximation: the average *E(x)* (black dot, top horizontal axis) of the distribution of body sizes (in grey, bottom horizontal axis) leads through projection on the curve (vertical and horizontal grey arrows) to a value of survival, *p[E(x)]* that does not correspond to the average of the distribution of survival proportions, *E(p)*. The difference between *p[E(x)]* and *E(p)* is then accounted for by the second‐ and third‐order approximations, *E(p)*_*Taylor*_ (Figure 2d).

We calculated the three different approximations of the average survival probability by plugging the average, variance and third‐order moment of body size into the appropriate terms of Equation 3 (Figure 2e). For instance, for the mean‐based approximation, the value of average body size was plugged into the function *f(x)*_*j*_ and other terms of Equation 3 were set to zero. For Taylor 2nd and 3rd, we first differentiated *f(x)*_*j*_ three times, rearranging terms and obtained the recursive formula:

(5a) $$f^{\prime \prime \prime }\left(x\right)=k_{l}∙\left\{f^{\prime \prime }\left(x\right)∙\left[1-2f\left(x\right)\right]-2{\left[f^{\prime }\left(x\right)\right]}^{2}\right\}$$

(5b) $$f^{\prime \prime }\left(x\right)=k_{l}∙f^{\prime }\left(x\right)∙\left[1-2f\left(x\right)\right]$$

(5c) $$f^{\prime }\left(x\right)=k_{l}∙f\left(x\right)∙\left[1-f\left(x\right)\right]$$

In Equations 5a–c, the subscript *j* was omitted for simplicity, but one such second and third derivative was obtained per each quadrat *j*. We then plugged in each equation the average value of body size as *x*, as needed to obtain each approximation (Figure 2d). Those estimations were compared against the expected survival probability, calculated by averaging the fitted values of survival for the quadrat‐specific model. Comparisons were made using the so‐called Jensen gap, that is, the deviation between the above three approximations and the expected survival from the model (Figure 2e). Positive deviations denote situations when ignoring body size variation results in an overestimation of survival, while negative values will indicate underestimation.

## 3 RESULTS

### 3.1 Effects of size and density

**FIGURE 3:**
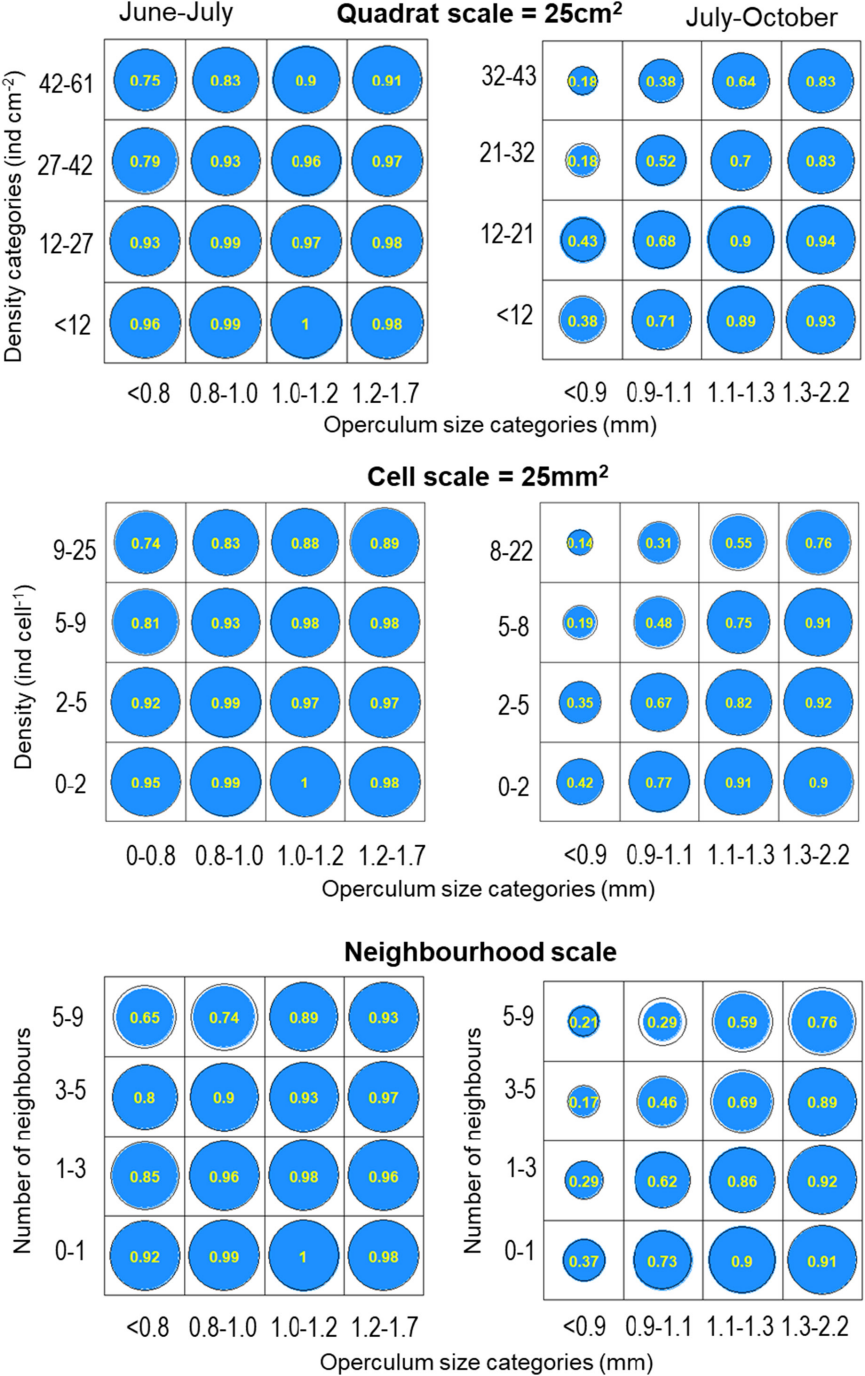
Shore 1. Bivariate distribution of proportion of survivors (numbers and size of blue circles) binned in size and density (or number neighbours) classes. Those classes were defined as percentiles: 0%–25%, 25%–50%, 50%–75% and 75%–100%. Each panel correspond to a period and scale of observation (quadrat, cell and neighbourhood). Black circular contours correspond to the value predicted from statistical models at mid‐quantiles: 12.5%, 37.5%, 62.5 and 87.5%.

**FIGURE 4:**
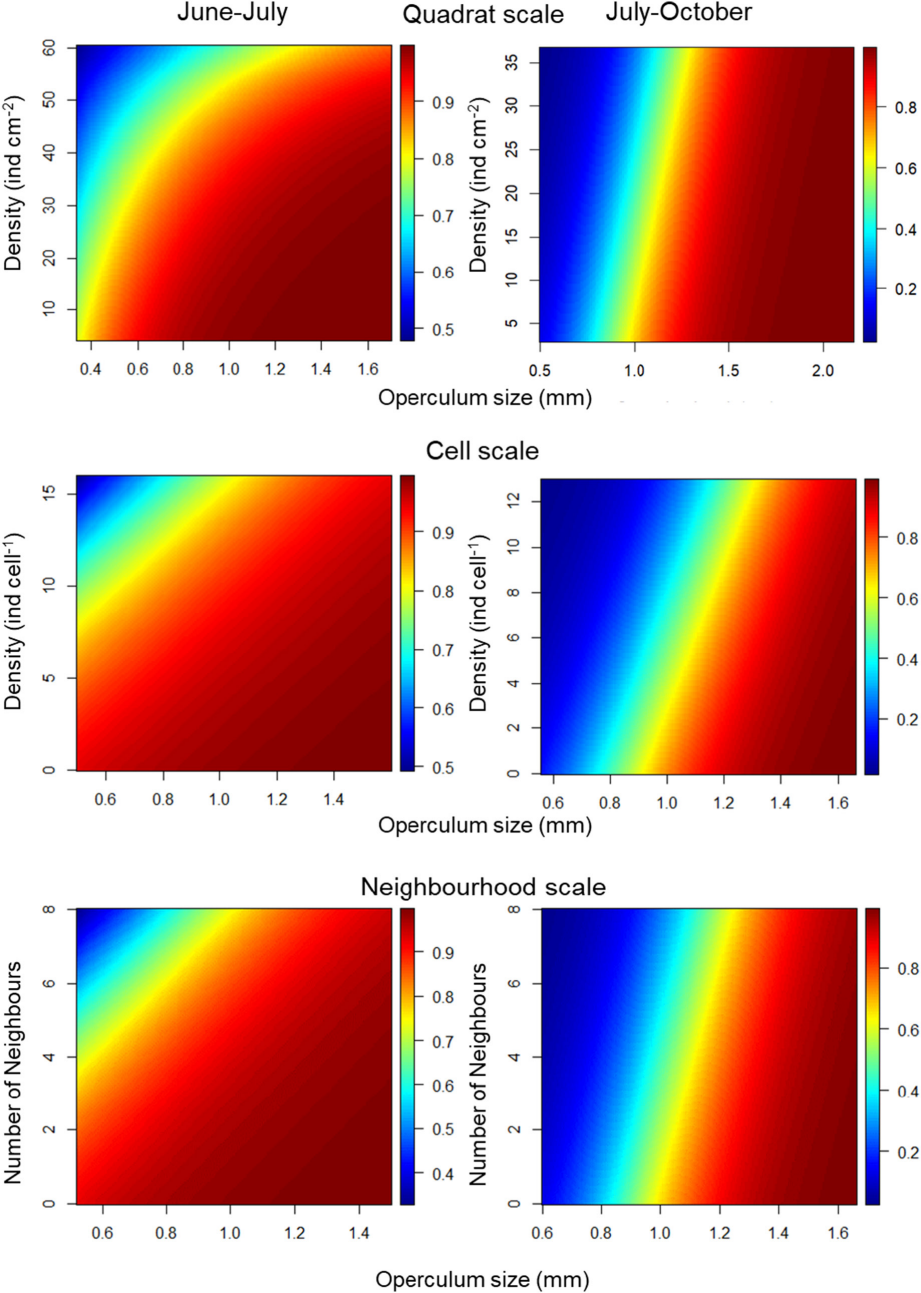
Predicted survival proportion in response to body size and density (or number of neighbours) at the scale of quadrats, cells or the neighbourhood around each target barnacle. Each panel corresponds to a period and scale of observation (quadrat: 25 cm^2^, cell: 25 mm^2^, neighbourhood).

The proportion of survivors (Figure 3a, see Figure S6 for shore 2) increased with barnacle size and decreased with barnacle density at all scales of observation especially between July and October. Between June and July, survival was consistently high and driven mainly by body size: best models for shore 1 (Figure 4, Table 1) showed a weak effect of size and density while the best models fitted for shore 2 (Figure S7) retained barnacle size as the only predictor. Between July and October, survival of the smaller body size fraction was strongly affected by barnacle density: here, best models retained both size and density as predictors, mostly operating additively on the logistic scale. For shore 1, survival between July and October was explained by models incorporating the number of exoskeletons of dead individuals. Effects of density and body size were consistent across scales in that most of the models selected coincided in structure; the only exception was June–July for shore 1 where the best model was interactive for the quadrat scale but additive for the cell and neighbour scale.

**TABLE 1:** Model selection of generalised linear models explaining juvenile survival rates in response to barnacle density (D) and body size (S; operculum length), for two time intervals in 2002 (June–July and July–August) and at two different shores in the west coast of the United Kingdom.

Model	Shore 1			Shore 2		
	June–July			June–July		
	Quadrat	Cell	Neigh	Quadrat	Cell	Neigh
S:D	**587**	545	580	399	388	393
S + D	590	**544**	**578**	397	386	391
D	662	566	601	445	432	440
S	625	566	620	**398**	**384**	**391**
Null	695	591	628	449	431	438
July–August				July–August		
Model	Quadrat	Cell	Neigh.	Quadrat	Cell	Neigh.
S:M		1103	1173		832	844
S + M		1101	**1171**		**830**	**842**
S:D	1212	1104	1180	858	837	846
**S + D**	1211	**1102**	1178	**856**	835	845
D	1280	1417	1502	1111	1087	1105
S	1530	1140	1183	881	851	845
Null	1611	1469	1502	1162	1111	1103

*Note*: Models were fitted with unstandardised response variables. Best models are highlighted in bold.

When models are additive in the logistic scale, they become interactive in the original scale because of the non‐linear effect of the logistic function. In our case, they showed a synergistic pattern in that the density‐dependent effect was more important in small than in large barnacles. This is consistent with the observed proportion of survival estimated at all scales: for example, in shore 1 at the cell scale (25 mm^2^), survival of the smaller body size fraction (first quartile) decreased by half (from 56% to 23%) from low to high densities while the survival of the largest size fraction decreased only slightly (from 98% to 87%).

**FIGURE 5:**
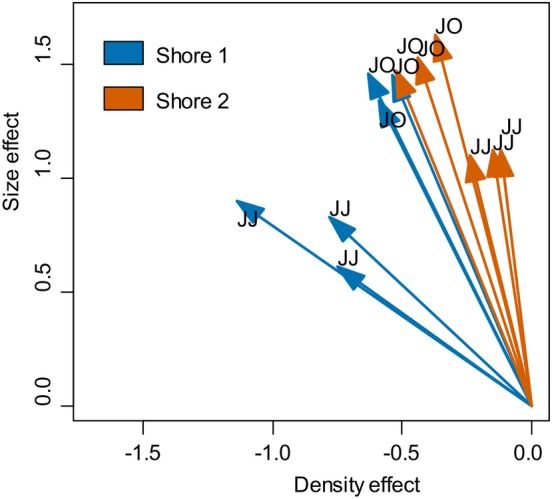
Vectorial representation of normalised parameter estimates, illustrating the importance of barnacle density and body size in driving survival. Parameter estimates were obtained by model fitting (see tables Sx and Sy for full values); for shore 2, the parameter estimates corresponding to the density effect did not differ significantly from zero. JJ, June–July; JO, July–October.

Parameter estimates (calculated from normalised predictors) showed comparable values, suggesting that (for the studied range of density and body size) natural variation in barnacle body size can be as important as barnacle density as a predictor of survival (Figure 5, Table 2). Calculation of explained variance also highlighted the importance of body size in explaining survival. For June–July, shores differed in the relative contribution of density and size‐dependent mortality: at shore 1, density contributed much more than in shore 2 where parameter estimates did not differ from zero. However, such differences disappeared in July–October when body density and size contributed to explaining barnacle survival.

**TABLE 2:** Relative contribution of body size and barnacle density to predict barnacle survival, as per cent of explained deviance of additive statistical models S + D (S = size, D = barnacle density or number of neighbours) at different spatial scales.

	Explained deviance (%)			
	June–July		July–August	
	Size	Density	Size	Density
Shore 1				
Quadrat	5.3	10.3	20.0	5.1
Cell	1.8	2.9	34.9	2.1
Neighbourhood	4.6	3.5	31.8	2.6
Shore 2				
Quadrat	11.1	1.1	22.1	4.5
Cell	5.4	0	28.3	2.7
Neighbourhood	5.4	0	29.9	0.2

*Note*: At the quadrat scale, calculations were based on the deviances of the separate terms which sum coincided in most cases with the total explained deviance. For the cell and neighbourhood scale, the values correspond to the marginal *R* ^2^ calculated from Stoffel et al. (2021).

### 3.2 Jensen gap

**FIGURE 6:**
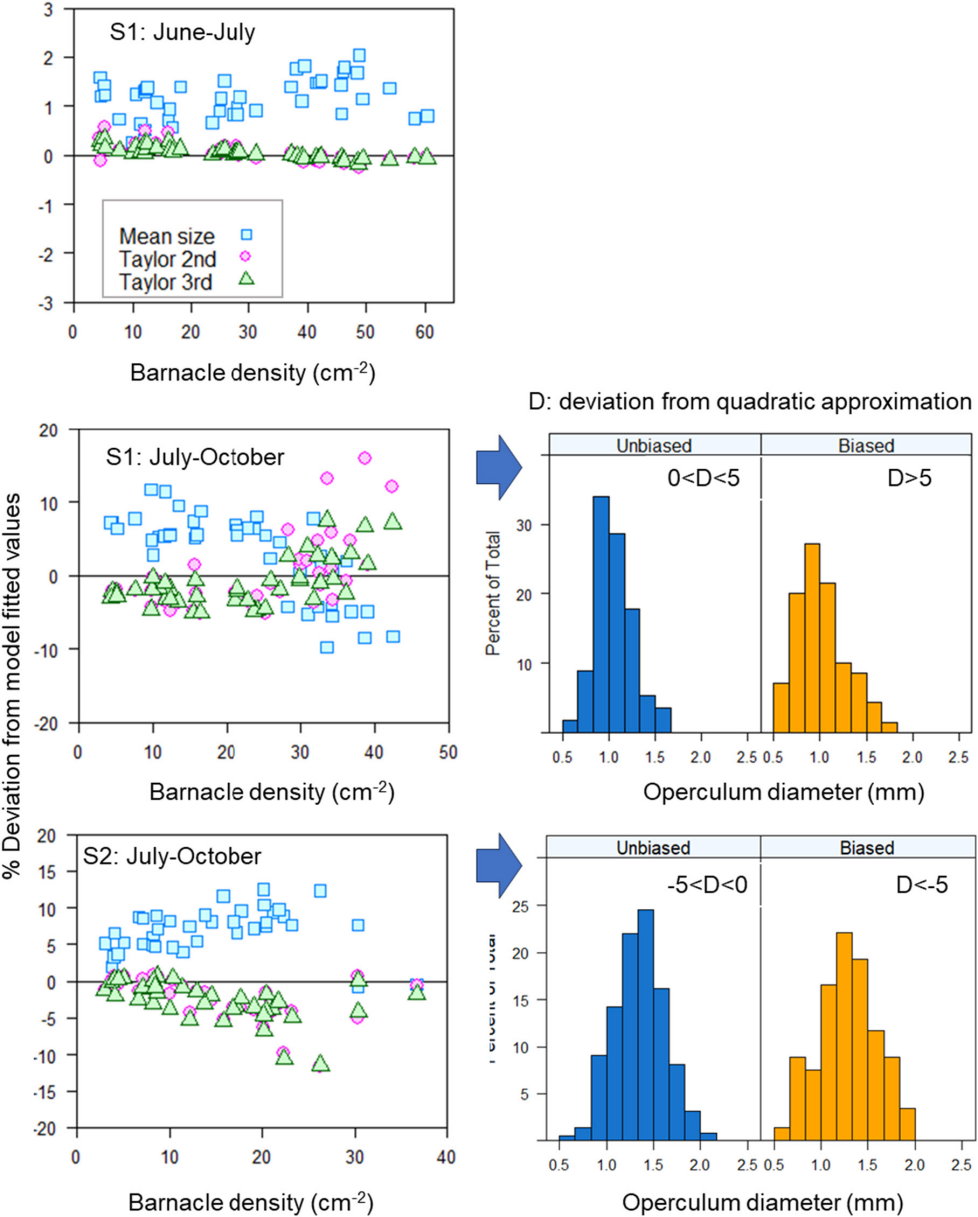
The Jensen gap: left panels: deviation from model fitted values for three estimations of the expected proportional survival, at each barnacle density for each shore and time period (shore 2–June–July not shown because the best model did not contain barnacle density). The expected proportional survival was calculated (1) for an average‐sized barnacle (2) based on second‐order Taylor approximation around the mean size, that is, up to the variance term and up to the third‐order moment which reflects the skewness of the distribution. Positive deviations: Situations indicate overestimation of survival; negative values indicate underestimation. Right panels: Size distribution of barnacles for cases of small and large biases (positive and negative deviations).

The quantification of the Jensen gap (quadrat scale only) led to over‐ or underestimation of the survival response when body size variation was ignored (Figure 6); bias in estimation of survival was higher at barnacle densities where size‐dependent survival was more important. At low barnacle densities and for June–July (shore 1), ignorance of body size variation resulted in very small bias (<5%). By contrast, at high barnacle densities, there were inaccuracies in survival estimation which varied depending on the shore and time period analysed. There was no evidence of a consistent bias in estimation since shores differed in whether the bias was positive or negative. The incorporation of the variance and skewness in size distribution (Figure 6) considerably reduced the size of the Jensen gap, especially for June–July but resulted in less success for July–October; however, the incorporation of skewness reduced the bias for several quadrats in shore 1.

A comparison of size distributions of barnacles according to the different levels of bias highlighted the importance of bimodal size distributions in the populations. From June to July at shore 1, the bias at high densities switched from negative to positive once the second‐ and third‐order approximations were applied. For those quadrats where the deviation of the quadratic approximation was <5%, the barnacle size distribution had a mode at 0.9–1.0 mm and very low proportion of either small or large barnacles; however, for the quadrats with a deviation >5%, the right tail of the distribution shows a large proportion of large‐sized barnacles. For shore 2, the patterns of deviation of the quadratic approximation were opposite to those of shore 1: here, the deviation switched from positive to negative once the second‐ and third‐order approximations were applied. Again, the size distribution in quadrats with deviation near zero (−5%–0%) showed a clear peak (1.3–1.5 mm) and unimodal distribution with low proportions of large‐ and small‐sized barnacles. However, those with negative deviation (< −5%) had, in addition to the main mode, a second mode indicating a large proportion of small‐sized barnacles.

## 4 DISCUSSION

We found evidence that spatial intraspecific variation in body size, within a cohort or individuals settling in the same month, can be as important as spatial variation in density in predicting survival of the marine barnacle *S. balanoides*. At all spatial scales examined, body size had a modulating effect on density‐dependent survival with larger individuals experiencing less negative effects of density than the smaller ones. In fact for large barnacles, density‐dependent mortality was practically irrelevant, while for smaller individuals, survival decreased from >80% to <50% along the natural range of densities found in the studied shores. The effect of body size on survival fluctuated through time; they were stronger as animals grew, presumably increasing the contact rate with neighbours. Our results combined with those of Wethey's (1983) give evidence in favour of a paramount role of size as a driver of population dynamics of *S. balanoides* across shores of different continents and contributes to understand the causes of spatial variation in the dynamics of open populations. Current theory is based on numerical effects, that is, on how population size is driven by the number of individuals colonising the adult habitat (recruitment limited populations) versus subsequent density‐dependent mortality (recruitment regulation:Caley et al., 1996; Hixon et al., 2002) but it is clear that the incorporation of spatial variation in body size should increase our understanding of processes driving open populations.

The effects of body size variation on survival were reflected in the Jensen Gap which was wider at higher barnacle densities. Theory of non‐linear averaging predicts that body size variation reduces population growth rates because a local population with individual variation in survival probabilities should have lower mean survival compared to a homogeneous population (Bolnick et al., 2011; Fox & Kendall, 2002). Our calculations suggest that in the field, the situation is more complex. The Jensen Gap depends not only on the curvature of the function linking body size and survival but also on the amount of variation in body size, which in itself can vary along the gradient of population density. We identified two different forms of variation based on whether the Jensen gap was negative or positive (i.e. quantified as deviations of the second‐order approximation). Small absolute deviations (i.e. near zero) were associated with unimodal size distributions with short tails. Positive deviation indicated the existence of additional cohorts of large individuals while negative deviation reflected additional cohorts of small individuals (as compared to the mode). Hence, under field conditions, the type of bias will therefore depend on the size distribution of individuals, and the scale transition approach may provide appropriate estimates of survival. Our analysis shows that information of the size distribution of individuals (along with that provided with the curvature survival function) might help ecologists to infer if they are over‐ or underestimating average responses.

The quantification of the contribution of body size in explaining survival relative to that of barnacle density requires an unbiased estimation of body size distributions. Typical approaches used to randomly select a subset of individuals include the point intercept method; an important consequence of such method is that the probability of an individual being sampled increases with the area of substratum covered (Zvuloni & Belmaker, 2016), which scales with the square of the body length. The undersampling of the smallest size classes and oversampling of the largest ones would result in a large amount of the size‐dependent effect being removed from the survival data (see e.g. Figure 3).

The current theory of open populations predicts outcomes along gradients defined by disturbance, recruitment and density‐dependent mortality (Caley et al., 1996; Hixon et al., 2002). However, unexpected patterns of population density may be driven by intraspecific trait variation. For instance, resistance to disturbances appears to be enhanced by trait diversity (presumably driven by genetic diversity: Hughes & Stachowicz, 2004). In oysters, marine taxa that like barnacles are characterised by complex life cycles, trait diversity of adults can drive colonisation by propagules (Smee et al., 2013) and survival at low predator density (Hanley et al., 2016), while body size diversity can drive cohort survival (Gribben et al., 2020). Our study suggests that further unexpected patterns may arise depending on three main characteristics: (1) the effect of body size on density‐dependent mortality, (2) the spatial correlation between body size and density and (3) the nature of the effect of body size on survival. Given the effect of body size on density‐dependent survival found in our study, we expect that size‐dependent mortality should be high in areas of high barnacle settlement. The modulation effect produced by body size should be scale‐dependent because density‐dependent processes vary at several spatial scales (Gaines & Roughgarden, 1985; Johnson, 2006; Schmitt & Holbrook, 2007), particularly for *S. balanoides* (Jenkins et al., 2000).

The degree of spatial correlation between body size and density is likely to be another important factor driving survival at the scale of the metapopulation. For example, in *S. balanoides*, body size varies at scales of 100s of km (e.g. along West Scotland: Burrows et al., 2010); hence, variation in body size may drive regional‐scale variation in survival, depending on the relationship with barnacle density. If spatial patterns of body size are not correlated with those of density, both small and large barnacles should coexist just after settlement, at both high and low barnacle densities. Hence, in that case, our findings predict that body size should drive post‐metamorphic survival in areas with high densities. By contrast, if spatial patterns of body size and density are correlated, the role of body size should be lower because of greater similarity of body sizes among neighbours.

The third important point is the nature of the effect of body size on survival. Our results are consistent with ‘the bigger the better’ hypothesis (Fontes et al., 2011; Green & McCormick, 2005; Marshall et al., 2006). In *S. balanoides*, large barnacles are likely to outcompete smaller individuals through a dominance suppression effect (Wethey, 1983) by crushing the shells or by limiting access to food. Hence, the role of body size as a modulator of density dependence in *S. balanoides* is similar to the one found in plants in search of light (Aarssen, 1995; White & Harper, 1970) and in other invertebrates (Marshall et al., 2006). Upward growth in *S. balanoides* is driven by the necessity to capture food and oxygen. However, the outcome of the diversity of body size across a metapopulation may depend on additional factors. For example, body size may interact with other factors such as genetic richness (e.g. bivalves: Hedge et al., 2014). Body size richness may provide an associational refuge from predators (Gribben et al., 2020), resulting in higher survival of smaller individuals at sites characterised by a diversity of body sizes.

The mechanism by which size variation is generated is central to understanding the dynamics of open populations. Size variation may arise through both pre‐ and post‐settlement processes interacting with genetic variation. Pre‐settlement processes should drive variation in the timing of settlement; those include (1) differences in timing of larval release and the rate of pelagic development, (2) variation in the time at which larvae are delivered to the rocky shore by currents and (3) differences in larval quality at settlement, driven by, for example, larval nutritional conditions (Emlet & Sadro, 2006; Jarrett, 2003; Torres et al., 2016). Variable growth post‐settlement may drive size variation if there is sufficient spatial heterogeneity in the drivers of juvenile growth (e.g. temperature and food availability: Sanford et al., 1994). Post‐settlement growth then results in a peak in the strength of density‐dependent survival (Gimenez & Jenkins, 2013; Jenkins et al., 2008) which is critical for small recruits (this study).

Overall, we conclude that intraspecific variation in body size, within a cohort, can be as important as density in driving survival of juvenile barnacles *S. balanoides*. This finding along with others calls for a revision of theory of open populations; failing to consider the role of body size variation, quantified through the Jensen gap, can be large especially when cohort size distribution is bimodal. Instead, theory of open populations should advance from simultaneous evaluation of the interacting effect of population density and body size, their spatial coupling and the specific role in driving survival. Quantification of ITV at multiple scales is needed, following the same logic used to quantify scale‐dependent variation in ecological processes (Chave, 2013; Levin, 1992; Wiens, 1989).

## AUTHOR CONTRIBUTIONS

**Luis Giménez:** Conceptualization (equal); data curation (supporting); formal analysis (lead); investigation (equal); methodology (equal); writing – original draft (lead); writing – review and editing (equal). **Stuart R. Jenkins:** Conceptualization (equal); data curation (lead); formal analysis (supporting); investigation (equal); methodology (equal); writing – original draft (supporting); writing – review and editing (equal).

## FUNDING INFORMATION

None.

## CONFLICT OF INTEREST STATEMENT

None.

## Supporting information


Data S1.

## Data Availability

We would like to store the data in Dryad. In addition, we can provide the data and R‐script for analysis as parts of the Supplementary Material.

## References

[ece370065-bib-0001] Aarssen, L. W. (1995). Hypotheses for the evolution of apical dominance in plants: Implications for the interpretation of overcompensation. Oikos, 74, 149–156. 10.2307/3545684

[ece370065-bib-0002] Altwegg, R. , & Reyer, H. U. (2003). Patterns of natural selection on size at metamorphosis in water frogs. Evolution, 57, 872–882. 10.1111/j.0014-3820.2003.tb00298.x 12778556

[ece370065-bib-0003] Audzijonyte, A. , Kuparinen, A. , Gorton, R. , & Fulton, E. A. (2013). Ecological consequences of body size decline in harvested fish species: Positive feedback loops in trophic interactions amplify human impact. Biology Letters, 9, 20121103. 10.1098/rsbl.2012.1103 23365151 PMC3639762

[ece370065-bib-0004] Barnes, H. , & Powell, H. T. (1950). The development, general morphology and subsequent elimination of barnacle populations, *Balanus crenatus* and *B. Balanoides*, after a heavy initial settlement. The Journal of Animal Ecology, 19, 175–179. 10.2307/1526

[ece370065-bib-0005] Begon, M. , Towsend, C. R. , & Harper, J. L. (2006). Ecology: From individuals to ecosystems (4th ed.). Blackwell Scientific Publications.

[ece370065-bib-0006] Bertness, M. D. , Gaines, S. D. , & Yeh, S. M. (1998). Making mountains out of barnacles: The dynamics of acorn barnacle hummocking. Ecology, 79, 1382–1394. 10.1890/0012-9658(1998)079[1382:MMOOBT]2.0.CO;2

[ece370065-bib-0007] Bolnick, D. I. , Amarasekare, P. , Araujo, M. S. , Burger, R. , & Levine, J. M. (2011). Why intraspecific trait variation matters in community ecology. Trends in Ecology & Evolution, 26, 183–192. 10.1016/j.tree.2011.01.009 21367482 PMC3088364

[ece370065-bib-0008] Burrows, M. T. , Jenkins, S. R. , Robb, L. , & Harvey, R. (2010). Spatial variation in size and density of adult and post‐settlement *Semibalanus balanoides*: Effects of oceanographic and local conditions. Marine Ecology Progress Series, 398, 207–219. 10.3354/meps08340

[ece370065-bib-0009] Caley, M. J. , Carr, M. H. , Hixon, M. A. , Hughes, T. P. , Jones, G. P. , & Menge, B. A. (1996). Recruitment and the local dynamics of open marine populations. Annual Review of Ecology and Systematics, 27, 477. 10.1146/annurev.ecolsys.27.1.477

[ece370065-bib-0010] Cameron, H. , Coulson, T. , & Marshall, D. J. (2019). Size and density mediate transitions between competition and facilitation. Ecology Letters, 22, 1879–1888. 10.1111/ele.13381 31468661

[ece370065-bib-0011] Caswell, H. (2001). Matrix population models (p. 722). Sinauer.

[ece370065-bib-0012] Chave, J. (2013). The problem of pattern and scale in ecology: What have we learned in 20 years? Ecology Letters, 16, 4–16. 10.1111/ele.12048 23351093

[ece370065-bib-0013] Connell, J. H. (1961). The influence of interspecific competition and other factors on the distribution of the barnacle *Chthamalus stellatus* . Ecology, 42, 710–723. 10.2307/1933500

[ece370065-bib-0014] Crisp, D. J. (1960). Factors influencing growth‐rate in *Balanus balanoides* . The Journal of Animal Ecology, 29, 95–116. 10.2307/2273

[ece370065-bib-0015] Des Roches, S. , Post, D. M. , Turley, N. E. , Bailey, J. K. , Hendry, A. P. , Kinnison, M. T. , Schweitzer, J. A. , & Palkovacs, E. P. (2018). The ecological importance of intraspecific variation. Nature Ecology & Evolution, 2, 57–64. 10.1038/s41559-017-0402-5 29203921

[ece370065-bib-0016] Emlet, R. B. , & Sadro, S. S. (2006). Linking stages of life history: How larval quality translates into juvenile performance for an intertidal barnacle (*Balanus glandula*). Integrative and Comparative Biology, 46, 334–346. 10.1093/icb/icj023 21672746

[ece370065-bib-0017] Fontes, J. , Santos, R. S. , Afonso, P. , & Caselle, J. E. (2011). Larval growth, size, stage duration and recruitment success of a temperate reef fish. Journal of Sea Research, 65, 1–7. 10.1016/j.seares.2010.05.001

[ece370065-bib-0018] Fox, G. A. , & Kendall, B. E. (2002). Demographic stochasticity and the variance reduction effect. Ecology, 83, 1928–1934. 10.2307/3071775

[ece370065-bib-0019] Gaines, S. , & Roughgarden, J. (1985). Larval settlement rate: A leading determinant of structure in an ecological community of the marine intertidal zone. Proceedings of the National Academy of Sciences of the United States of America, 82, 3707–3711. 10.1073/pnas.82.11.3707 16593571 PMC397856

[ece370065-bib-0020] Gardner, J. L. , Peters, A. , Kearney, M. R. , Joseph, L. , & Heinsohn, R. (2011). Declining body size: A third universal response to warming? Trends in Ecology & Evolution, 26, 285–291. 21470708 10.1016/j.tree.2011.03.005

[ece370065-bib-0021] Gimenez, L. , & Jenkins, S. R. (2013). Combining traits and density to model recruitment of sessile organisms. PLoS One, 8, e57849. 10.1016/j.tree.2011.03.005 23469247 PMC3585730

[ece370065-bib-0022] Green, B. S. , & McCormick, M. I. (2005). Maternal and paternal effects determine size, growth and performance in larvae of a tropical reef fish. Marine Ecology Progress Series, 289, 263–272. 10.3354/meps289263

[ece370065-bib-0023] Gribben, P. , Bishop, M. J. , O'Connor, W. A. , Bradley, D. J. , & Hughes, A. R. (2020). Intraspecific diversity in prey body size influences survivorship by conferring resistance to predation. Ecosphere, 11, e03106.

[ece370065-bib-0024] Griffin, J. N. , & Silliman, B. R. (2018). Predator size‐structure and species identity determine cascading effects in a coastal ecosystem. Ecology and Evolution, 8, 12435–12442. 30619556 10.1002/ece3.4571 PMC6308854

[ece370065-bib-0025] Hanley, T. C. , Highes, R. A. , Williams, B. , Garland, H. , & Kimbro, D. L. (2016). Effects of intraspecific diversity on survivorship, growth, and recruitment of the eastern oyster across sites. Ecology, 97, 1518–1529. 27459782 10.1890/15-1710.1

[ece370065-bib-0026] Hartig, F. (2022). DHARMa: Residual diagnostics for hierarchical (multi‐level/mixed) regression models. R Package Version 0.4.6.

[ece370065-bib-0027] Hedge, L. H. , Leung, B. , O'Connor, W. A. , & Johnston, E. (2014). The interacting effects of diversity and propagule pressure on early colonization and population size. The Journal of Animal Ecology, 83, 168–175. 24001312 10.1111/1365-2656.12125

[ece370065-bib-0028] Hixon, M. A. , Pacala, S. W. , & Sandin, S. A. (2002). Population regulation: Historical context and contemporary challenges of open vs. closed systems. Ecology, 83, 1490–1508. 10.1890/0012-9658(2002)083[1490:PRHCAC]2.0.CO;2

[ece370065-bib-0029] Hughes, A. R. , & Stachowicz, J. J. (2004). Genetic diversity enhances the resistance of a seagrass ecosystem to disturbance. Proceedings of the National Academy of Sciences of the United States of America, 101, 8998–9002. 15184681 10.1073/pnas.0402642101 PMC428461

[ece370065-bib-0030] Jarrett, J. N. (2003). Seasonal variation in larval condition and postsettlement performance of the barnacle *Semibalanus balanoides* . Ecology, 84, 384–390. 10.1890/0012-9658(2003)084[0384:SVILCA]2.0.CO;2

[ece370065-bib-0031] Jenkins, S. R. , Aberg, P. , Cervin, G. , Coleman, R. A. , Delany, J. , Della Santina, P. , Hawkins, S. J. , LaCroix, E. , Myers, A. A. , Lindegarth, M. , & Power, A. M. (2000). Spatial and temporal variation in settlement and recruitment of the intertidal barnacle *Semibalanus balanoides* (L.) (crustacea: Cirripedia) over a European scale. Journal of Experimental Marine Biology and Ecology, 243, 209–225. 10.1016/S0022-0981(99)00121-5

[ece370065-bib-0032] Jenkins, S. R. , Murua, J. , & Burrows, M. T. (2008). Temporal changes in the strength of density‐dependent mortality and growth in intertidal barnacles. The Journal of Animal Ecology, 77, 573–584. 10.1111/j.1365-2656.2008.01366.x 18284479

[ece370065-bib-0033] Johnson, D. W. (2006). Density dependence in marine fish populations revealed at small and large spatial scales. Ecology, 87, 319–325. 10.1890/04-1665 16637357

[ece370065-bib-0034] Levin, S. A. (1992). The problem of pattern and scale in ecology: The robert H. MacArthur award lecture. Ecology, 73, 1943–1967. 10.2307/1941447

[ece370065-bib-0035] Lindmark, M. , Huss, M. , Ohlberger, J. , & Gårdmark, A. (2018). Temperature‐dependent body size effects determine population responses to climate warming. Ecology Letters, 21, 181–189. 10.1111/ele.12880 29161762

[ece370065-bib-0036] Marshall, D. J. , Cook, C. N. , & Emlet, R. B. (2006). Offspring size effects mediate competitive interactions in a colonial marine invertebrate. Ecology, 87, 214–225. 10.1890/05-0350 16634312

[ece370065-bib-0037] Marshall, D. J. , Pettersen, A. K. , & Hayley, C. (2018). A global synthesis of offspring size variation, its eco‐evolutionary causes and consequences. Functional Ecology, 32, 1436–1446. 10.1111/1365-2435.13099

[ece370065-bib-0038] Moran, E. V. , Hartig, F. , & Bell, D. M. (2016). Intraspecific trait variation across scales: Implications for understanding global change responses. Global Change Biology, 22, 137–150. 10.1111/gcb.13000 26061811

[ece370065-bib-0039] Nakagawa, S. , & Schielzeth, H. (2013). A general and simple method for obtaining R^2^ from generalized linear mixed‐effects models. Methods in Ecology and Evolution, 4, 133–142. 10.1111/j.2041-210x.2012.00261.x

[ece370065-bib-0040] Ohlberger, J. (2013). Climate warming and ectotherm body size–From individual physiology to community ecology. Functional Ecology, 27, 991–1001. 10.1111/1365-2435.12098

[ece370065-bib-0041] Ricklefs, R. E. , & Miller, G. L. (1999). Ecology. Freeman & Co Ltd.

[ece370065-bib-0042] Rowe, L. , & Ludwig, D. (1991). Size and timing of metamorphosis in complex life cycles: Time constraints and variation. Ecology, 72, 413–427. 10.2307/2937184

[ece370065-bib-0043] Sanford, E. , Bermudez, D. , Bertness, M. D. , & Gaines, S. D. (1994). Flow, food supply and acorn barnacle population dynamics. Marine Ecology Progress Series, 104, 49–62. https://www.int‐res.com/articles/meps/104/m104p049.pdf

[ece370065-bib-0044] Schmitt, R. J. , & Holbrook, S. J. (2007). The scale and cause of spatial heterogeneity in strength of temporal density dependence. Ecology, 88, 1241. 10.1890/06-0970 17536410

[ece370065-bib-0045] Smee, D. L. , Overath, D. O. , Johnson, K. D. , & Sanchez, J. A. (2013). Intraspecific variation influences natural settlement of eastern oysters. Oecologia, 173, 947–953. 23543216 10.1007/s00442-013-2645-4

[ece370065-bib-0046] Stoffel, M. A. , Nakagawa, S. , & Schielzeth, H. (2021). partR2: Partitioning R2 in generalized linear mixed models. PeerJ, 9, e11414. 10.7717/peerj.11414 34113487 PMC8162244

[ece370065-bib-0047] Stump, S. M. , Song, C. , Saavedra, S. , Levine, J. M. , & Vasseur, D. A. (2022). Synthesizing the effects of individual‐level variation on coexistence. Ecological Monographs, 92, e01493. 10.1002/ecm.1493

[ece370065-bib-0048] Torres, G. , Gimenez, L. , Pettersen, A. K. , Bue, M. , Burrows, M. T. , & Jenkins, S. R. (2016). Persistent and context‐dependent effects of the larval feeding environment on post‐metamorphic performance through the adult stage. Marine Ecology Progress Series, 545, 147–160. 10.3354/meps11586

[ece370065-bib-0049] Toscano, B. J. , & Griffen, B. D. (2012). Predatory crab size diversity and bivalve consumption in oyster reefs. Marine Ecology Progress Series, 445, 65–74. 10.3354/meps09461

[ece370065-bib-0050] Venables, W. N. , & Ripley, B. D. (2002). Modern applied statistics with S (p. 510). Springer.

[ece370065-bib-0051] Violle, C. , Enquist, B. J. , McGill, B. J. , Jiang, L. , Albert, C. c. H. , Hulshof, C. , Jung, V. , & Messier, J. (2012). The return of the variance: Intraspecific variability in community ecology. Trends in Ecology & Evolution, 27, 244–252. 10.1016/j.tree.2011.11.014 22244797

[ece370065-bib-0052] Wiens, J. A. (1989). Spatial scaling in ecology. Functional Ecology, 3, 385–397.

[ece370065-bib-0053] West‐Eberhard, M.‐J. (2003). Developmental plasticity and evolution (p. 814). Oxford University Press.

[ece370065-bib-0054] Wethey, D. S. (1983). Intrapopulation variation in growth of sessile organisms: Natural populations of the intertidal barnacle *Balanus balanoides* . Oikos, 40, 14–23. 10.2307/3544195

[ece370065-bib-0055] White, J. , & Harper, J. L. (1970). Correlated changes in plant size and number in plant populations. Journal of Ecology, 58, 467–485. 10.2307/2258284

[ece370065-bib-0056] Xu, M. (2016). Ecological scaling laws link individual body size variation to population abundance fluctuation. Oikos, 125, 288–299. 10.1111/oik.03100

[ece370065-bib-0057] Zaiats, A. , Germino, M. J. , Serpe, M. D. , Richardson, B. A. , & Caughlin, T. T. (2021). Intraspecific variation mediates density dependence in a genetically diverse plant species. Ecology, 102, e03502. 10.1002/ecy.3502 34314039

[ece370065-bib-0058] Zuur, A. F. , Ieno, E. N. , Walker, N. , Saveliev, A. A. , & Smith, G. M. (2009). Mixed effects models and extensions in ecology with R. Springer‐Verlag.

[ece370065-bib-0059] Zvuloni, A. , & Belmaker, J. (2016). Estimating ecological count‐based measures from the point‐intercept method. Marine Ecology Progress Series, 556, 123–130. 10.3354/meps11853

