# Correction to “The Role of Intraspecific Trait Variation in Driving Post‐Metamorphic Survival: Implications for Recruitment in Open Populations”

**DOI:** 10.1002/ece3.71602

**Published:** 2025-06-19

Giménez, L. and S. R. Jenkins. 2024. “The Role of Intraspecific Trait Variation in Driving Post‐Metamorphic Survival: Implications for Recruitment in Open Populations.” *Ecology and Evolution* 14: e70065. https://doi.org/10.1002/ece3.70065.

In Paragraph 5, in the section of Methods 2.3, “Jensen Gap” (below Equation 4), the following text is incorrect: “*dg*/*dx* = *a* + *b*∙*N*_*j*_ + *c* = *k*_1_” and “*dg*/*dx* = *a* + *b*∙*N*_*j*_ + *c* + *d*∙*N*_*j*_ = *k*_2_.” This should read as follows: “*dg*/*dx* = *c* = *k*_*1*_” and “*dg*/*dx* = *c* + *d N*_*j*_ = *k*_2_.”

We apologize for this error.

