# Supplementary material for: The role of intraspecific trait variation in driving post‐metamorphic survival: Implications for recruitment in open populations
Source: Ecol Evol. 2024 Aug 6;14(8):e70065. doi: 10.1002/ece3.70065 (PMC11301026; doi:10.1002/ece3.70065)
Supplement: Supplementary file 1 — Data S1. [file ECE3-14-e70065-s001.docx]

The role of Intraspecific trait variation in driving recruitment in OPEN POPULATIONs

**SUPPLEMENT**

The correlation between density predictors and delimitation of the predictor space used to fit statistical models.


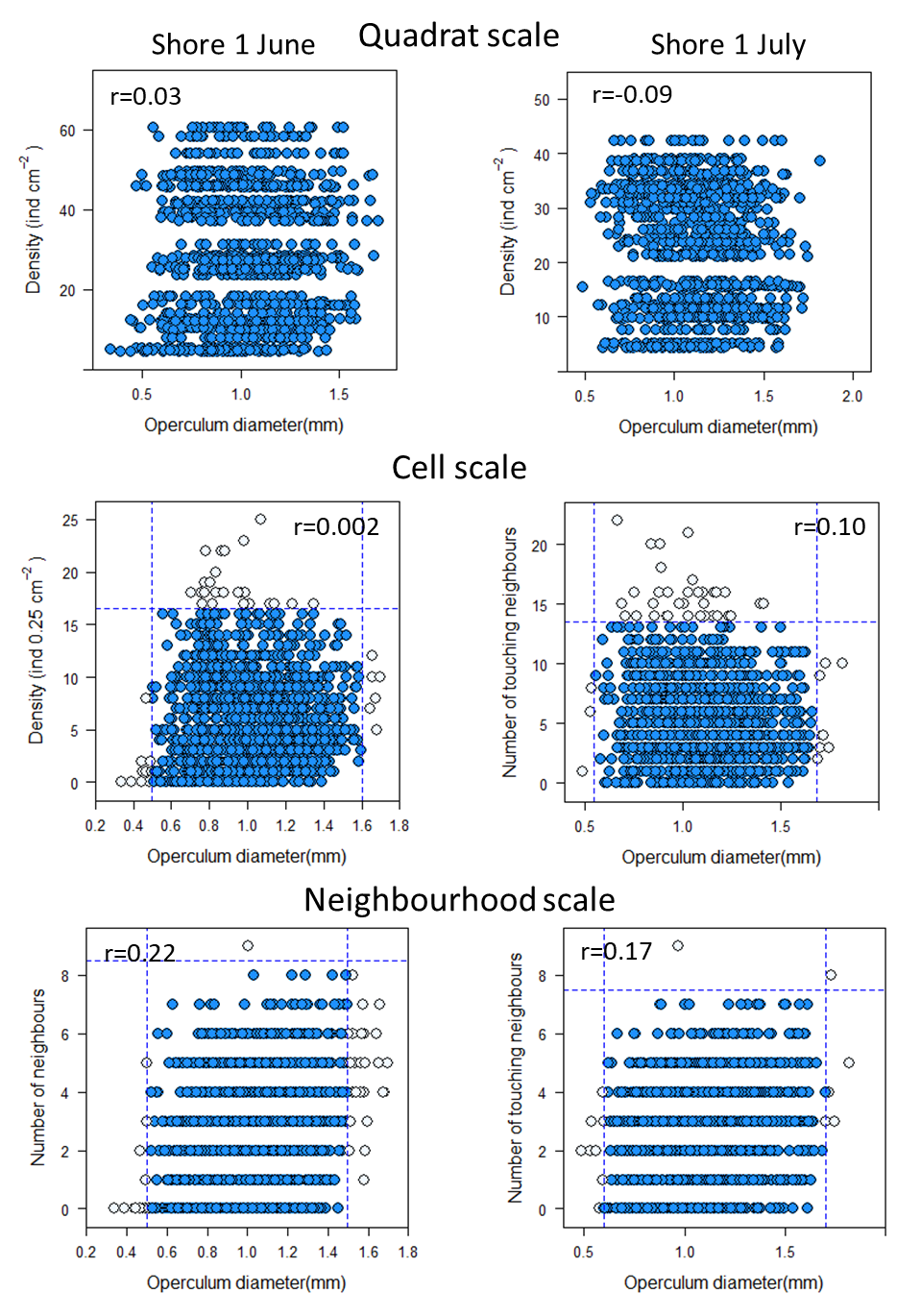


Figure S1. Shore 1. Distribution of data points in the predictor space defined by operculum size and barnacle density (or number of touching neighbours) for each spatial scale and time period (June: June-July; July: July-October).


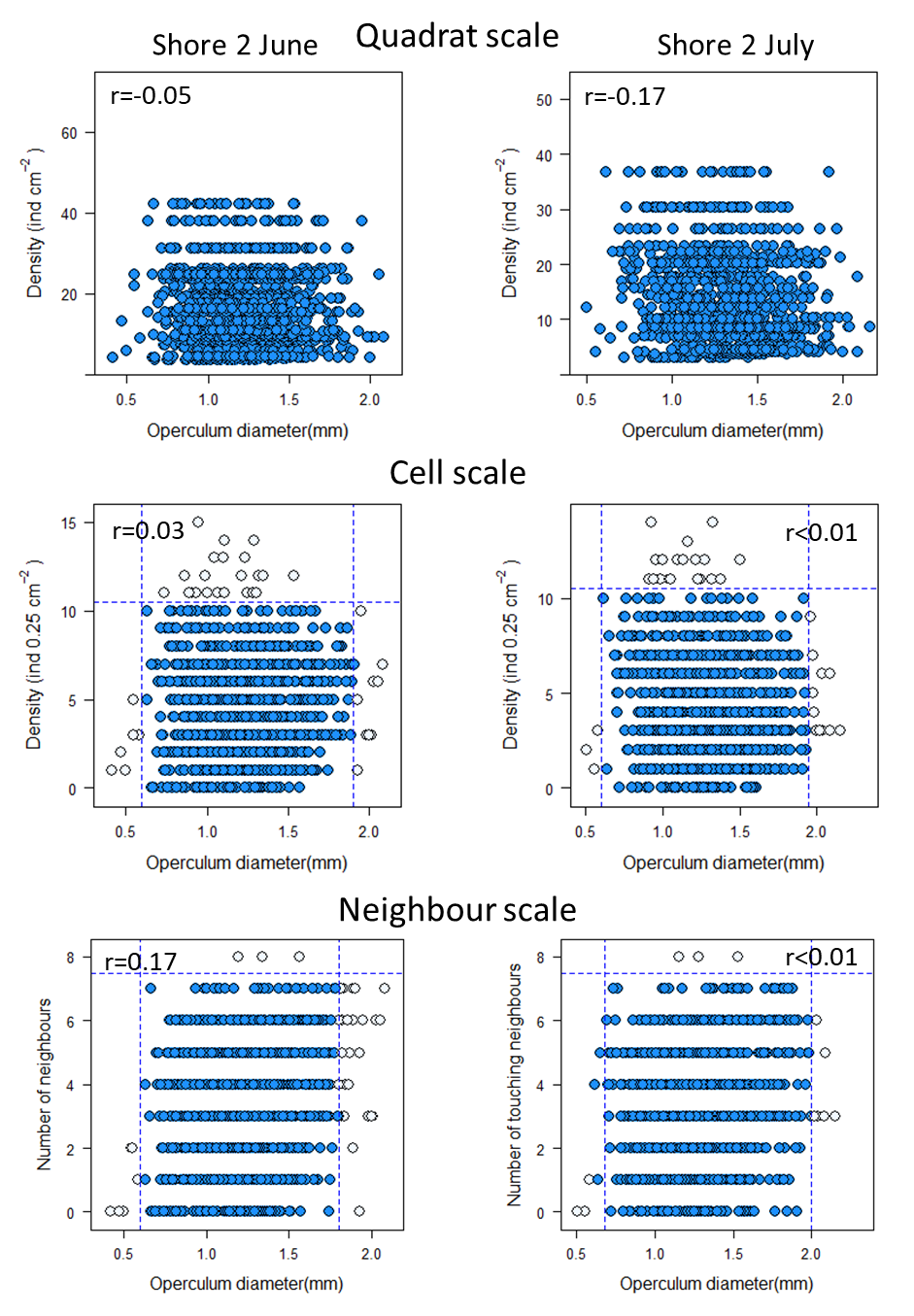


Figure S2. Shore 2. Distribution of data points in the predictor space defined by operculum size and barnacle density (or number of touching neighbours) for each spatial scale and time period (June: June-July; July: July-October).

2. Model Validation


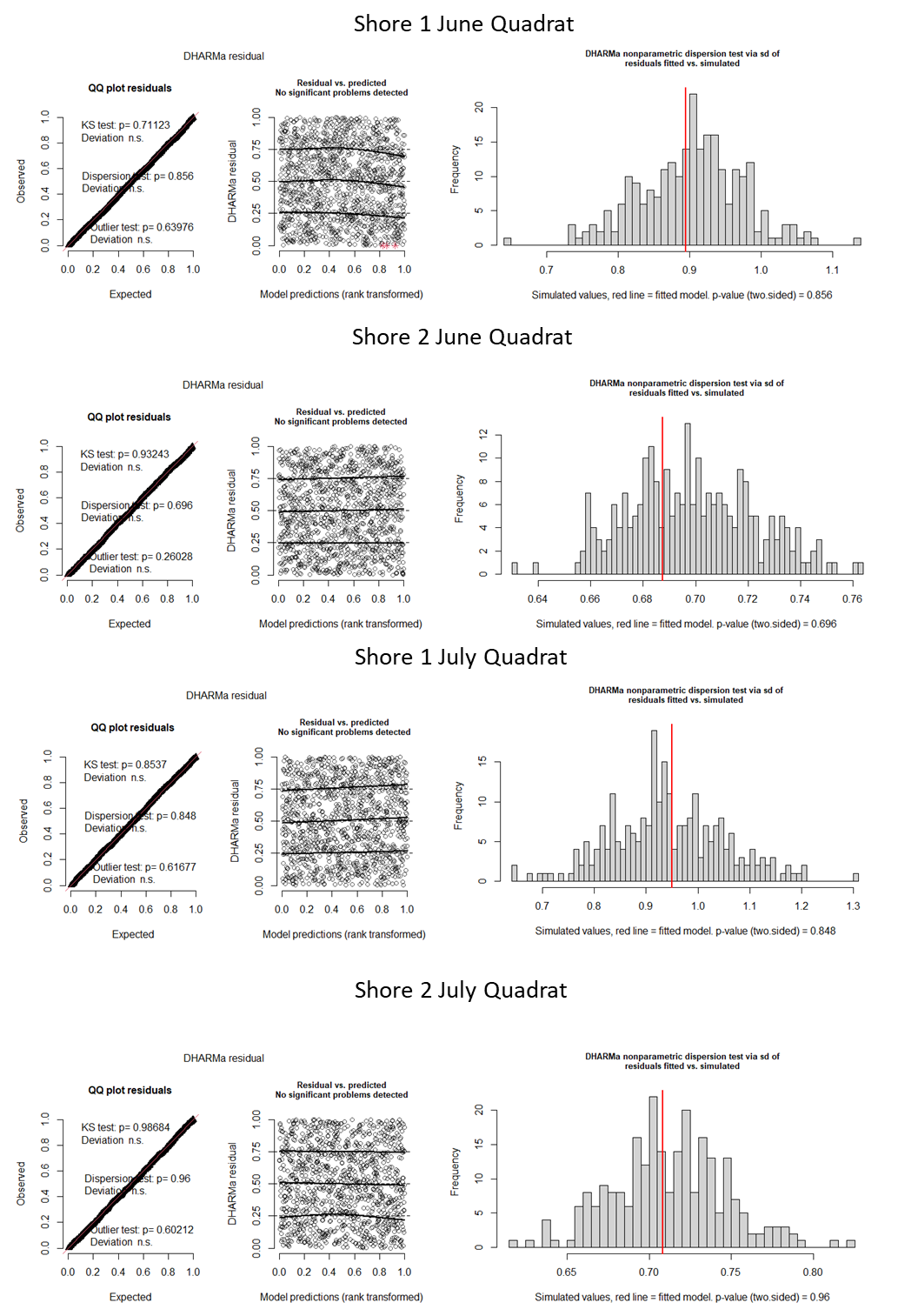


Figure S3. Analysis of residuals of model at the scale of the quadrat (25cm^2^) using the DHARMa package for the combinations of shore and time period. Left panels: qq-plot used to detect deviations from the expected distribution. Central panels: plot of residuals vs predicted values. Right panels: non-parametric dispersion tests comparing the fitted model (red line) with the distribution of simulated values (grey bars).


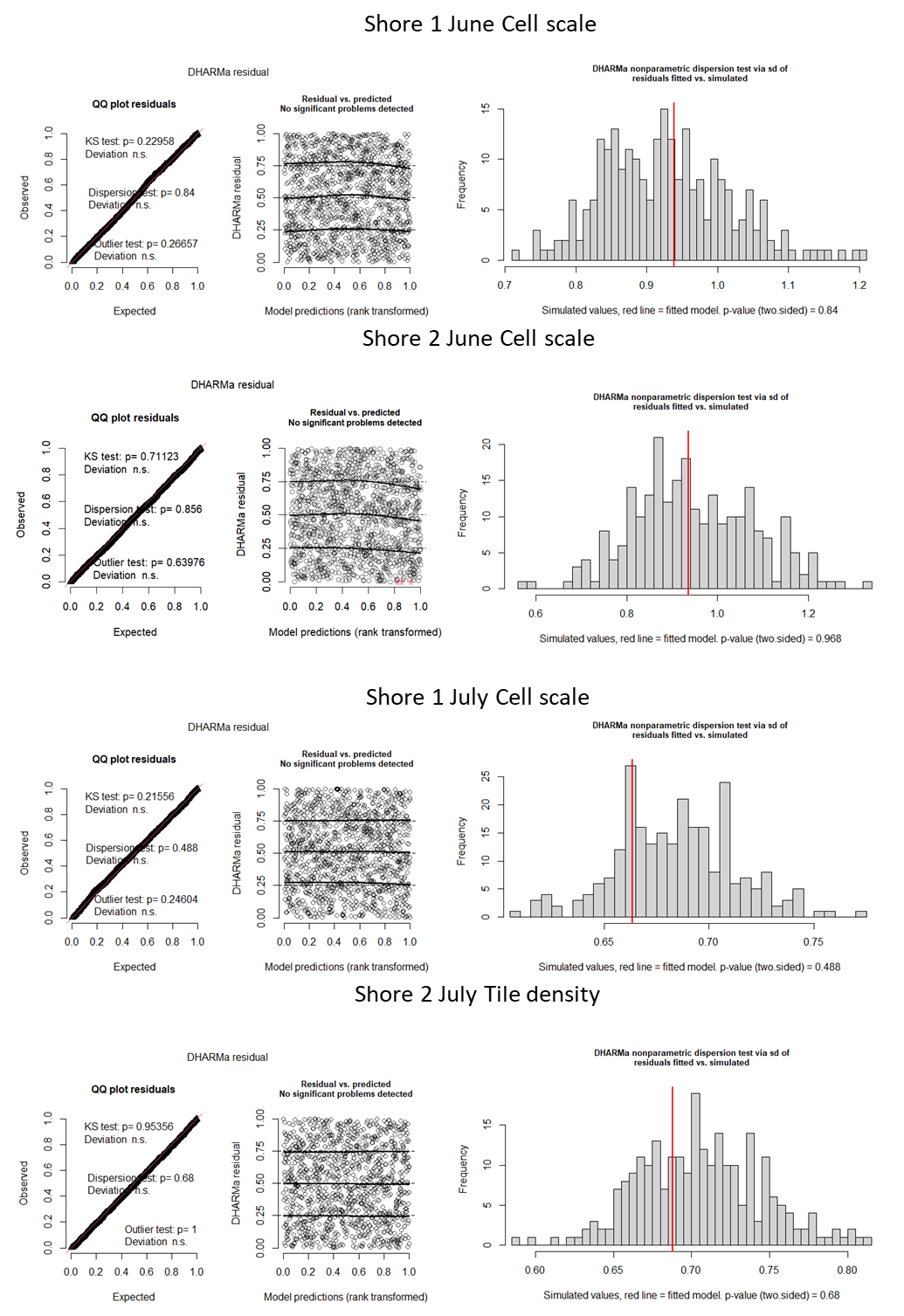


Figure S4. Analysis of residuals of model at the scale of a cell (25mm^2^) using the DHARMa package for the combinations of shore and time period. Left panels: qq-plot used to detect deviations from the expected distribution. Central panels: plot of residuals vs predicted values. Right panels: non-parametric dispersion tests comparing the fitted model (red line) with the distribution of simulated values (grey bars).


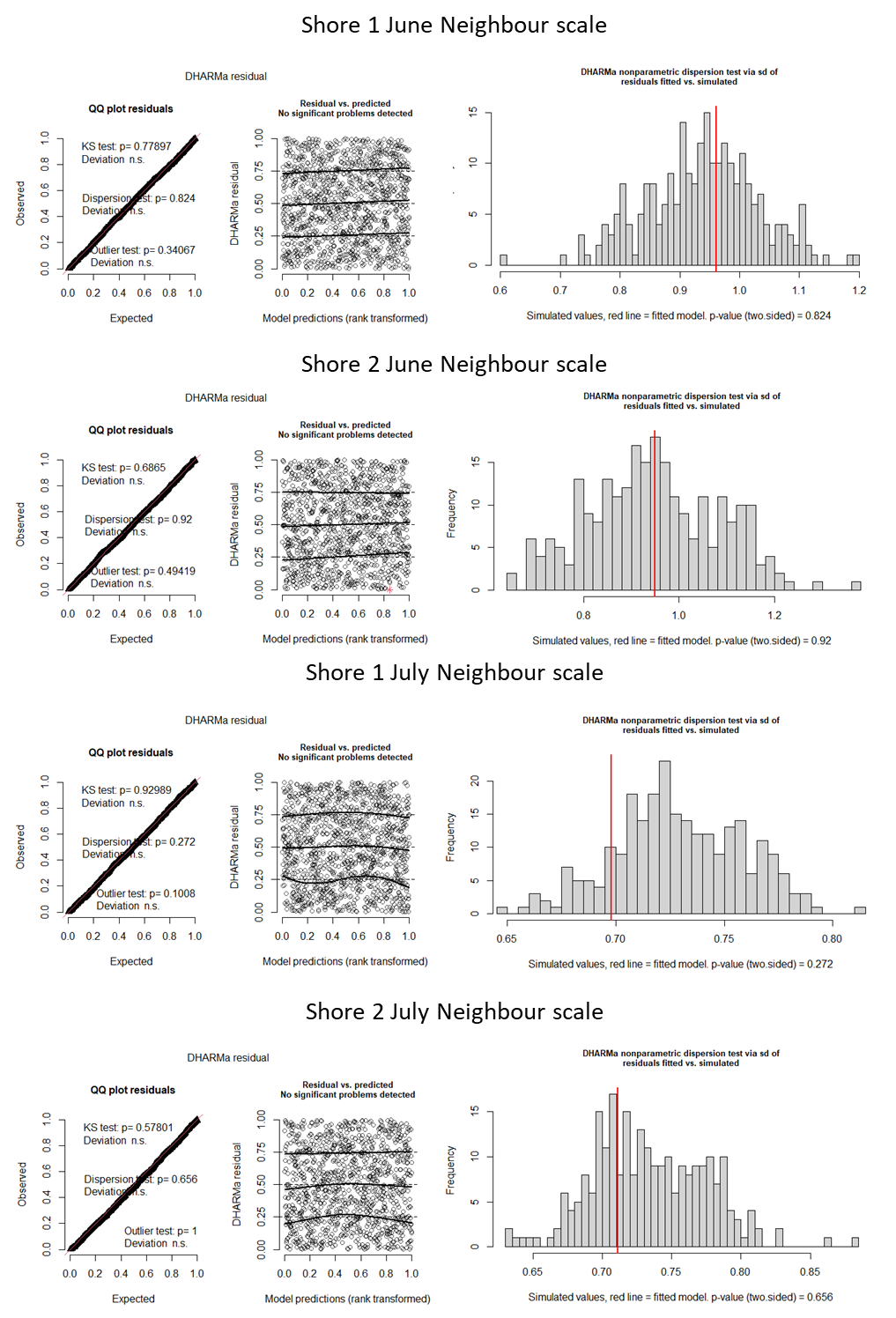


Figure S5. Analysis of residuals of model at the scale of the neighbourhood using the DHARMa package for the combinations of shore and time period. Left panels: qq-plot used to detect deviations from the expected distribution. Central panels: plot of residuals vs predicted values. Right panels: non-parametric dispersion tests comparing the fitted model (red line) with the distribution of simulated values (grey bars).


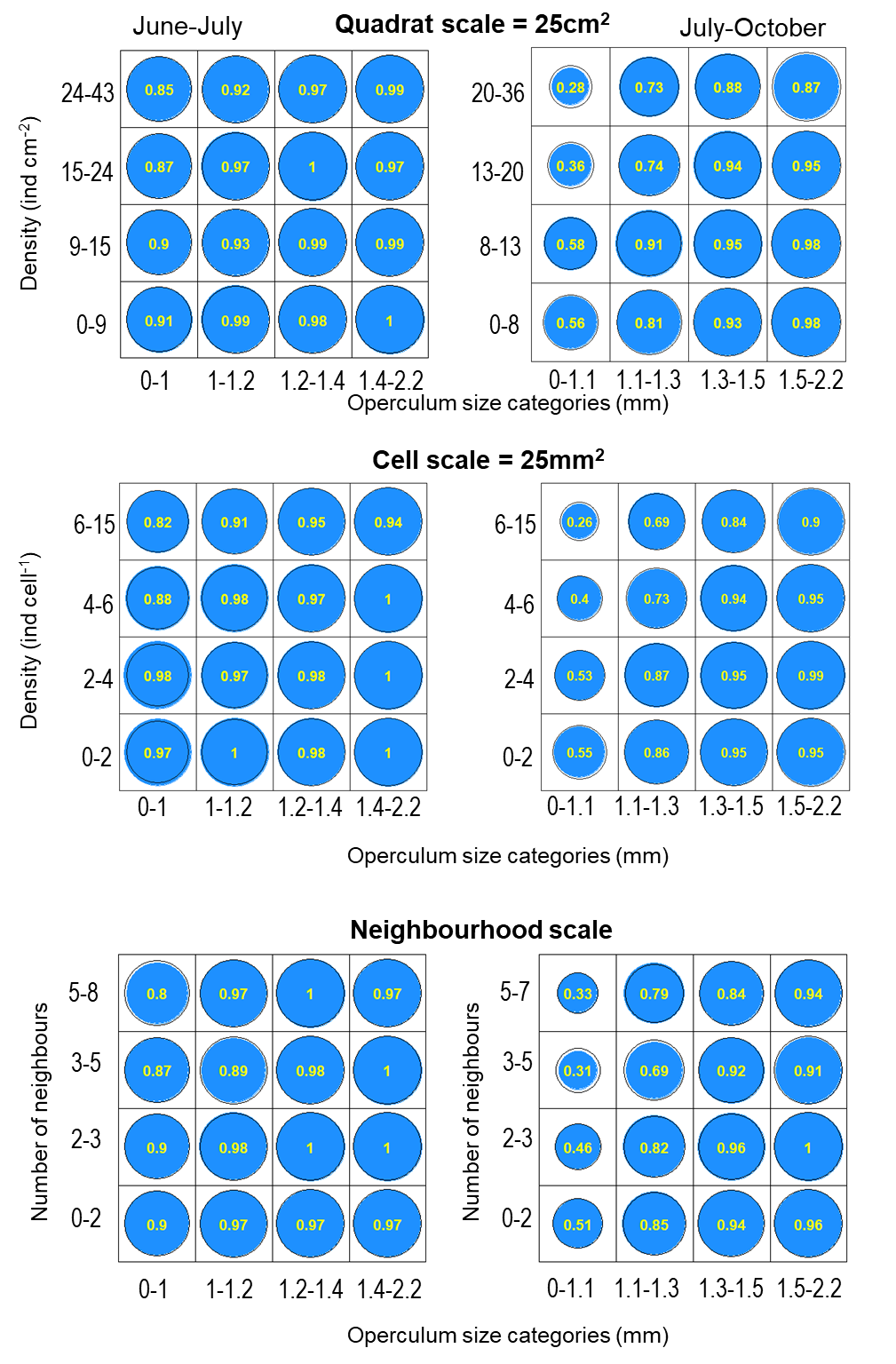


Figure S6. Shore 2. Bivariate distribution of proportion of survivors (numbers and size of blue circles) binned according to percentiles in four categories: 0-25%, 25-50% and 50-75% and 75-100% of the distribution of sizes and densities (or number of touching neighbours). Each panel correspond to a period and scale of observation (Tile: 25 cm^2^, Cell: 25 mm^2^, Neighbourhood). Black circular contours correspond to the value predicted from statistical models at mid quantiles: 12.5%, 37.5% and 62.5 and 87.5%.


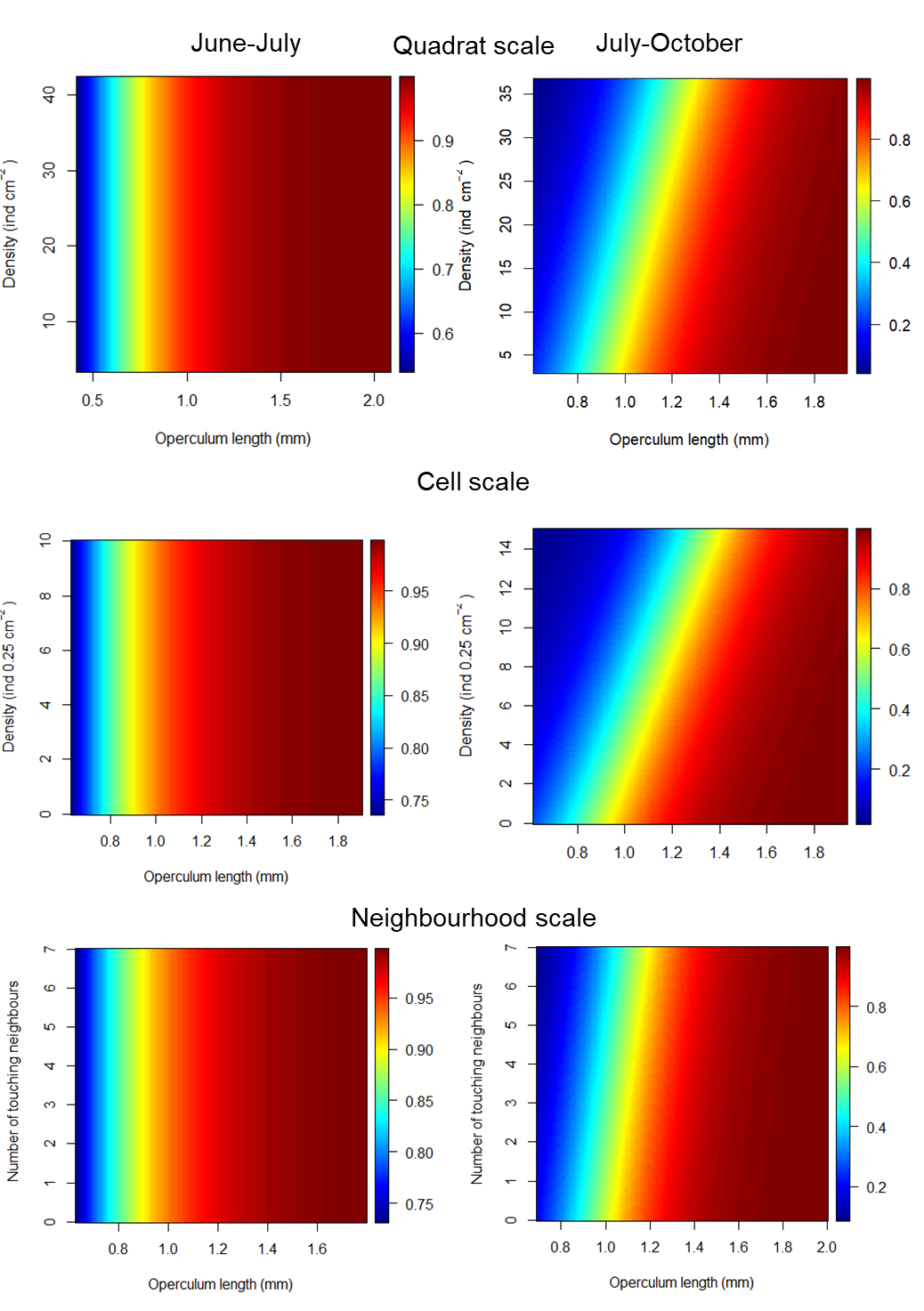


Figure S7. Predicted survival proportion in response to body size and density (or number of neighbours) at the scale of tiles, cells or the neighbourhood around each target barnacle. Each panel corresponds to a period and scale of observation (Tile: 25 cm^2^, Cell: 25 mm^2^, Neighbourhood).

Table S1. Shore 1: Parameter estimates of the best generalised linear models of survival rates in response to barnacle density and body size. Estimates are shown for normalised predictors (i.e. after centring and standardization by standard deviation) that are unitless. In such case the parameter estimates represent proportional increases by a unit of standard deviation. Barnacles were sampled at two shores and time periods in 2002 (June-July and July-August). Most of the best model containing both density and size were additive in the logistic scale.

| Shore 1 | June-July | | July-August | |
| --- | --- | --- | --- | --- |
| Tile scale | Estimate | SE | Estimate | SE |
| Intercept | 3.15 | 0.19 | 0.76 | 0.07 |
| Density | -1.14 | 0.16 | -0.59 | 0.07 |
| Size | 0.90 | 0.15 | 1.33 | 0.09 |
| Size x Density | -0.26 | 0.12 |  |  |
| Cell scale |  |  |  |  |
| Intercept | 3.16 | 0.18 | 0.86 | 0.12 |
| Density | -0.75 | 0.13 | -0.63 | 0.10 |
| Size | 0.61 | 0.13 | 1.46 | 0.10 |
| Neighbour scale |  |  |  |  |
| Intercept | 3.15 | 0.19 | 0.81 | 0.07 |
| Number | -0.78 | 0.15 | -0.39 | 0.07 |
| Size | 0.83 | 0.13 | 1.50 | 0.09 |

Table S2. Shore 2: Parameter estimates of the best generalised linear models of survival rates in response to barnacle density and body size. Estimates are shown for normalised predictors (i.e. after centring and standardization by standard deviation) that are unitless. In such case the parameter estimates represent proportional increases by a unit of standard deviation. Barnacles were sampled at two shores and time periods in 2002 (June-July and July-August). Most of the best model containing both density and size were additive in the logistic scale.

| Shore 2 | June-July | | July-August | |
| --- | --- | --- | --- | --- |
| Tile scale | Estimate | SE | Estimate | SE |
| Intercept | 3.49 | 0.20 | 1.78 | 0.10 |
| Density | -0.24 | 0.13 | -0.44 | 0.08 |
| Size | 1.10 | 0.17 | 1.53 | 0.11 |
| Cell scale |  |  |  |  |
| Intercept | 3.60 | 0.25 | 1.76 | 0.11 |
| Density | -0.12 | 0.16 | -0.52 | 0.08 |
| Size | 1.12 | 0.18 | 1.47 | 0.11 |
| Neighbour scale |  |  |  |  |
| Intercept | 3.57 | 0.25 | 1.84 | 0.15 |
| Density | -0.15 | 0.16 | -0.25 | 0.11 |
| Size | 1.12 | 0.18 | 1.69 | 0.13 |
